# *TREM-1* SNP rs2234246 regulates TREM-1 protein and mRNA levels and is associated with plasma levels of L-selectin

**DOI:** 10.1371/journal.pone.0182226

**Published:** 2017-08-03

**Authors:** Alex-Ander Aldasoro Arguinano, Sébastien Dadé, Maria Stathopoulou, Marc Derive, Ndeye Coumba Ndiaye, Ting Xie, Christine Masson, Sébastien Gibot, Sophie Visvikis-Siest

**Affiliations:** 1 UMR INSERM U1122; IGE-PCV ‘Interactions Gène-Environnement en Physiopathologie Cardiovasculaire’, Faculté de Pharmacie–Université de Lorraine, Nancy, France; 2 INOTREM, Nancy, France; 3 Réanimation Médicale, Hôpital Central, Nancy, France; 4 Department of Internal Medicine and Geriatrics, CHU Technopôle Nancy-Brabois, Rue du Morvan, Vandoeuvre-lès-Nancy, France; Institut national de la recherche scientifique, CANADA

## Abstract

High levels of TREM-1 are associated with cardiovascular and inflammatory diseases risks and the most recent studies have showed that *TREM-1* deletion or blockade is associated with up to 60% reduction of the development of atherosclerosis. So far, it is unknown whether the levels of TREM-1 protein are genetically regulated. Moreover, TREM family receptors have been suggested to regulate the cellular adhesion process. The goal of this study was to investigate whether polymorphisms within *TREM-1* are regulating the variants of serum TREM-1 levels and the expression levels of their mRNA. Furthermore, we aimed to point out associations between polymorphisms on *TREM-1* and blood levels of selectins. Among the 10 SNPs studied, the minor allele T of rs2234246, was associated with increased sTREM-1 in the discovery population (p-value = 0.003), explaining 33% of its variance, and with increased levels of mRNA (p-value = 0.007). The same allele was associated with increased soluble L-selectin levels (p-value = 0.011). The higher levels of sTREM-1 and L-selectin were confirmed in the replication population (p-value = 0.0007 and p-value = 0.018 respectively). We demonstrated for the first time one SNP on *TREM-1*, affecting its expression levels. These novel results, support the hypothesis that TREM-1 affects monocytes extravasation and accumulation processes leading to atherogenesis and atherosclerotic plaque progression, possibly through increased inflammation and subsequent higher expression of sL-selectin.

## Introduction

Atherosclerosis is a multifactorial disease affecting arterial blood vessels due to a chronic inflammatory response. One of the early stages of atherosclerosis is the endothelial adhesion/infiltration of white blood cells to the arterial sites, attracted by different pro-inflammatory molecules [1, 2]. This accumulative process gradually promotes inflammation and, by doing so, accelerates the atherosclerotic progression.

Triggering Receptor Expressed on Myeloid cells-1 (TREM-1) is a member of the recently discovered immunoglobulin family [3, 4]. It increases the inflammatory responses of the neutrophils and monocytes [5] and amplifies the production of pro-inflammatory cytokines (TNFα and IL-1beta) [6]. Furthermore, it has been reported that migrating neutrophils bind to TREM-1 [7], suggesting an important role of this receptor in the recruitment and mobilization of neutrophils and monocytes to the arterial intima [8]. Although TREM-1 was initially characterized for its role during the pathophysiology of septic shock [3], there are today increasing evidences that it is also implicated in other acute and chronic inflammatory diseases of non-infectious etiology, such as rheumatoid arthritis [9], atherosclerosis [10], acute myocardial infarction [11] and critical limb ischemia [12]. Activation of innate immunity and inflammatory cells recruitment and extravasation is a common feature of these diseases in which TREM-1 seems to be a central player [8, 10, 13]. Also, the serum levels of soluble TREM-1 (sTREM-1) correlate with the severity of these diseases [8–10, 12, 14–18]. Moreover, the genetic invalidation or pharmacological blockage of TREM-1 results in a reduced inflammatory state and improved outcome in animal models [19, 20]. The mechanistic process by which TREM-1 participates in inflammatory cell extravasation during inflammatory diseases remains to be clarified. It is well known the important role that the adhesion molecules, and specially the selectins, play during this specific process [21–23]. During the last two decades, the importance of the adhesion molecules in the process of inflammatory cells adhesion and trans-endothelial migration, which contributes to pathological inflammation and thrombosis in many preclinical diseases including atherosclerosis, has been widely studied [21, 24–26]. Thus, TREM-1 could be in close connection with the selectins.

TREM-1 gene polymorphisms have been associated with the development of acute inflammation and sepsis, but also with coronary artery disease. Indeed, the polymorphism rs4711668, which is located within the *TREM-1* gene has been associated with severe coronary atherosclerosis in a Russian population [27]. However, it is still unknown whether the levels of sTREM-1 are genetically regulated, and no polymorphism has been yet reported to affect its expression levels. Furthermore, another interesting part of TREM-1 regulation is that although there exists a specific splicing variant for the soluble form of the protein (TREM-1sv) [28], some authors argue that the mechanism of shedding of the membrane bound TREM-1 by metalloproteases is the main contributor for the release of sTREM-1 [7, 29].

In the present study, we investigated whether single nucleotide polymorphisms (SNPs) within or near the *TREM-1* gene were associated with soluble TREM-1 (sTREM-1 and TREM-1sv) serum levels and with the expression levels of two TREM-1 splicing isoforms, more precisely, the membrane form (mbTREM-1) and the soluble form (TREM-1sv). Moreover, we investigated the associations between the significant in the above associations SNPs and levels of soluble E-selectin, L-selectin and P-selectin (sE-selectin, sL-selectin and sP-selectin), which are involved in the early stages of atherosclerotic processes.

## Material and methods

### Ethics statement

The samples are part of a human sample storage platform: the Biological Resources Centre ‘Interactions Gène- Environnement en Physiopathologie CardioVasculaire’ (BRC IGE-PCV—number BB-0033-00051) in Nancy, East of France. All participants gave a written informed consent. All the populations involved in this study were recruited in accordance with the latest version of the Declaration of Helsinki for Ethical Principles for Medical Research Involving Human Subjects. All the protocols were approved by the local ethics committees for the protection of subjects for biomedical research: the Comité Consultatif de Protection des Persones dans la Recherche Biomédicale (CCPPRB).

### Study populations

The population enrolled in this study makes part of the STANISLAS Family Study (SFS) [30]. Participants were of French origin and were apparently in good health, not under lipid-lowering and/or cardiovascular drug treatment and free from chronic diseases. This cohort is a longitudinal family study designed to investigate factors related to cardio-vascular disease. The clinical data of the investigated individuals were obtained at the Centre for Preventive Medicine (CMP) of Vandoeuvre-lés-Nancy. Participants were of European descent and came from the Vosges and the South of Meurthe-et-Moselle, in the East of France. Among them, 30 unrelated individuals were used as discovery cohort for the selection of the *TREM-1* SNPs and for their relation with TREM-1 levels. A population of 351 unrelated individuals was used as discovery cohort for the associations with the adhesion molecules levels.

An independent population (n = 80), available in the Biological Resources Centre ‘Interactions Gène- Environnement en Physiopathologie CardioVasculaire’ (BRC IGE-PCV, number BB-0033-00051), composed of unrelated adults of French origin was used as replication population for the results of associations of SNPs with TREM-1 and adhesion molecules levels.

During and after the data collection, authors had access to information that could identify individual participants.

### Data collection and biological measurements

Body mass index (BMI) was measured using the Quetelet’s formula: weight divided by height squared (kg/m^2^). Blood samples were taken from the individuals after an overnight fast. Plasma and serum samples for adhesion molecules and TREM-1 measurements were frozen at -80°C until analysis.

Plasma levels of E-selectin, L-selectin and P-selectin were measured with enzyme-linked immunosorbent assay (R&D Systems, Abington, UK). Serum levels of soluble TREM-1 were measured with a double antibody sandwich ELISA assay (Quantikine Human TREM-1 Immunoassay ELISA Kit; R&D Systems, Minneapolis, MN, USA) using the iMARK Microplate Absorbance Reader (Bio-Rad).

All molecules were measured in duplicate and according to manufacturers’ instructions.

TREM-1sv levels were measured using a home-made ELISA assay adapted from Duo-Set ELISA assay (R&D Systems, hTREM-1 Duo-Set ELISA assay) and using a monoclonal anti-TREM-1sv antibody (R&D Systems) as capture antibody. A TREM-1sv recombinant protein was used as standard protein for quantification [28]. The sensitivity of the detection of TREM-1sv was of 15pg/mL.

### SNPs selection–Bioinformatics analyses

A bibliographic search of the SNPs within or near *TREM-1* was performed. The HuGE navigator (https://phgkb.cdc.gov/PHGKB/phgHome.action?action=home) and the NCBI dbSNP database (http://www.ncbi.nlm.nih.gov/projects/SNP/) were used for the selection of SNPs for the further investigations. Only SNPs with a minor allele frequency >5% according to HapMap were selected.

To point out if the significant SNPs are located in regulation zones (promoter, enhancer, silencers) of the *TREM-1* gene and are linked to specific epigenetic profiles (acetylation/methylation), bioinformatics analyses were performed comparing different cell types.

The SNPs were localised on the Human genome (GRCh38.p7) using Ensembl browser [31]. Identification of potent regulation zones and establishment of epigenetic profiles according to cell types, were determined using also this browser. Regulation and epigenetic profiles have been obtained using 23 different cell types from 19 healthy and 4 cancerous individuals from Ensembl release 87.

The possible influence of the SNPs on the transcription factor binding site (TFBS) was investigated by using the transcription factor database TRANSFAC R.3.4 [32].

### Genotyping

DNA was extracted from all participants, and relative biobanks have been constructed inside the BRC IGE-PCV. The SNPs were genotyped by Genoscreen (http://genoscreen.fr), using a Sequenom iPLEX Gold assay-Medium Throughput Genotyping Technology.

### Gene expression investigations

Gene expression analysis was performed on peripheral blood mononuclear cells (PBMCs) of a subsample of 30 individuals from the SFS. PBMCs were isolated by centrifugation on a density gradient of Ficoll. The RNA was extracted from PBMCs with the MagNAPure automate, using the MagNA Pure LC RNA HP isolation kit protocol (Roche Diagnostics, France) and the RNA quality was measured by the NanoDrop 1000 Spectrophotometer (Thermo Scientific). Total RNA was used to generate first-strand complementary DNA (cDNA) with the C1000 Thermal cycler (Bio-Rad) and the cDNA Synthesis Kit (Bio-Rad). The reactions conditions were as follows: 30 minutes at 42°C, followed by 5 minutes at 85°C.

The mbTREM-1 and the TREM-1sv were investigated. The primers used for the amplification of mbTREM-1 were as follows: forward primer, 5’-GTGACCAAGGGTTTTTCAGG-3’; reverse primer, 5’-ACACCGGAACCCTGATGATA-3’. The primers used for the amplification of TREM-1sv were as follows: forward primer, 5’-GTGGTGACCAAGGGGTTC-3’; reverse primer, 5’-AGATGGATGTGGCTGGAAGT-3’. The reaction conditions were as follows: Initial denaturation of 94°C for 2 min, followed by 40 cycles of denaturation at 94°C for 30 seconds, annealing at 58°C for 20 seconds, extension at 70°C for 20 seconds. For the absolute quantification of the 2 splicing forms of *TREM-1*, corresponding mRNA levels were normalized to the mRNA levels of beta 2 microglobulin (B2M) gene. The housekeeping gene was simultaneously amplified to check the RNA integrity and to verify that the same amounts of template were used in all cases. The absolute quantification of the 2 splicing forms of the *TREM-1* and the housekeeping gene were performed using Lightcycler Carousel-Based System (Roche Diagnostics) and SYBR Green I reaction Kit. Negative controls were used for each reaction and the specificity of the products was confirmed by polyacrylamide gel electrophoresis as well as by melt curve analysis. All experiments were performed in duplicate.

No PBMCs were available for the replication population and therefore this analysis was performed only in the SFS individuals.

### Statistical analyses

All investigated molecules (intermediate phenotypes) were log transformed in order to normalise their distribution before analyses. The normality of distribution was tested by Kolmogorov-Smirnov test. Hardy-Weinberg equilibrium was tested using the chi-square test. The SNPs-mRNA and soluble TREM-1 levels associations were assessed through linear regression adjusted for age, gender and BMI under three inheritance models (additive, dominant and recessive) and using the minor allele as the reference allele. The association of the most significant SNPs with selectins levels were tested using similar models. Association studies were performed using the Plink Software [33]. Populations’ characteristics were determined using the SPSS statistical software version 20.0 (SPSS, Inc, Chicago, Illinois). Bonferroni correction was applied in order to adjust the multiplicity of tests and avoid the type 1 error. Cut-offs were 0.05/ number of SNPs for the association of the selected SNPs with soluble TREM-1 levels, 0.05/ number of SNPs/2 for the associations of the significant SNPs with the 2 mRNA levels and 0.05/number of SNPs/number of selectins for the associations of the significant SNPs with the selectins’ levels.

## Results

HuGE navigator and NCBI dbSNP database searches resulted to the selection of 10 SNPs located within or near *TREM-1* with a minor allele frequency >5%. The 10 SNPs were genotyped in 30 individuals from the SFS. No significant deviation from Hardy-Weinberg equilibrium was observed for the studied polymorphisms (Table 1).

**Table 1:** Characteristics of the polymorphisms studied in 30 individuals of SFS.

SNP	Minor allele	Minor allele frequency (MAF)	Chromosome	HWE P
**rs3789204**	A	0.265	6	0.667
**rs7768162**	A	0.317	6	0.912
**rs7772334**	T	0.460	6	0.948
**rs728488**	T	0.368	6	0.505
**rs612399**	C	0.377	6	0.989
**rs2234246**	T	0.416	6	0.763
**rs2234237**	A	0.086	6	0.632
**rs13211886**	T	0.089	6	0.350
**rs6910730**	G	0.098	6	0.825
**rs9471554**	C	0.313	6	0.425

Demographic and clinical characteristics of the studied individuals are summarized in the Table 2.

### Association studies between the 10 polymorphisms in *TREM-1* and serum levels of sTREM-1/TREM-1sv

For this first decisional step, we used a discovery and a replication population. Demographic and clinical characteristics of the studied individuals included in each population are summarized in the Table 2.

**Table 2:** Demographic and clinical characteristics of the individuals.

	SFS sub-sample	SFS Total	Replication Population
**Sample size [% female]**	30 [47%]	351 [48.43%]	80 [52.5%]
**Age (years) [S.D]**	47.07 [5.3]	44.09 [4.3]	47 [8.1]
**BMI (kg/m^2^) [S.D]**	26 [4.69]	24.96 [3.76]	26.7 [3.69]
**sTREM-1 (pg/ml) [S.D]**	213.47 [70.24]	-	352.38 [87.88]
**TREM-1sv (pg/ml) [S.D]**	**-**	**-**	39.08 [32.01]
**L-selectin (mg/l) [S.D]**	948.68 [293.2]	1018.65 [280.8]	888.19 [74.67]
**P-selectin (mg/l) [S.D]**	132 [31.94]	137.93 [43.45]	-
**E-selectin (mg/l) [S.D]**	44.93 [11.68]	53.56 [27.07]	-
**CRP (mg/L) [S.D]**	1.41 [1.45]	1.83 [2.95]	-

**Fig 1:**
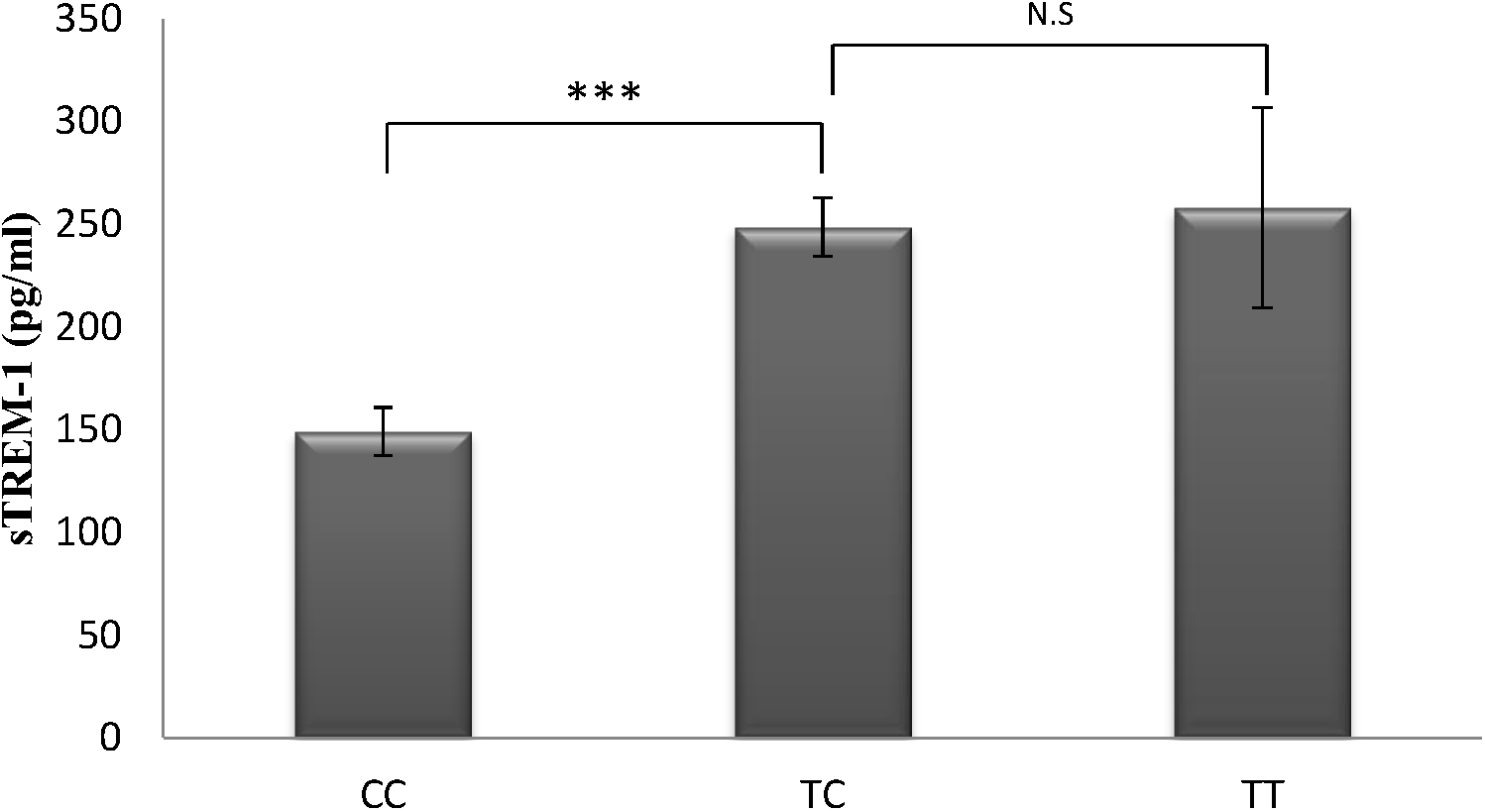
Mean values of sTREM-1 levels according to the different genotypes of rs2234246 (CC vs TC vs TT) in the discovery population. Thin bars show standard errors. CC; Homozygous for the major allele of the rs2234246. TC; Heterozygous for the rs2234246. TT; Homozygous for the minor allele of the rs2234246. The significance between genotypes is showed as follows; N.S.; >0.05, * p<0.05, ** p<0.01, *** p<0.001.

Association analyses were performed in 30 individuals of SFS (discovery population) for the 10 SNPs and the levels of TREM-1 in serum using the additive, dominant and recessive inheritance models (P-value cut-off was set at 0.05/10 = 0.005). Only for rs2234246 we found significant results. According to the additive model, the minor allele T of rs2234246 was associated with increased levels of sTREM-1 (p-value = 0.003, β = 0.3, data available in Table 3 and Fig 1). When the dominant model was used, this association was even stronger (p-value = 0.0003, β = 0.49), and the variance in the serum TREM-1 levels explained by the model is of 33% in the discovery population.

**Table 3:** Statistical associations of the 10 polymorphisms studied with the serum levels of TREM-1 and mRNA levels according to the different inheritance models. P-value thresholds for TREM-1 protein levels are P<0.005 for the discovery population and P<0.05 for the replication population. P-value threshold for mRNA levels is <0.025.

Polymorphisms	Model	sTREM-1 (protein) discovery (N = 30)			sTREM-1 (protein) replication (N = 80)			TREM-1sv (mRNA) (N = 30)			mbTREM-1 (mRNA) (N = 30)		
		P-value	β	S.E	P-value	β	S.E	P-value	β	S.E	P-value	β	S.E
rs3789204	Additive	0.090	0.25	0.14	-	-	-	-	-	-	-	-	-
	Dominant	0.090	0.25	0.14	-	-	-	-	-	-	-	-	-
	Recessive	-	-	-	-	-	-	-	-	-	-	-	-
rs7768162	Additive	0.024	0.21	0.08	-	-	-	-	-	-	-	-	-
	Dominant	0.037	0.28	0.12	-	-	-	-	-	-	-	-	-
	Recessive	0.139	0.28	0.18	-	-	-	-	-	-	-	-	-
rs7772334	Additive	0.105	-0.17	0.10	-	-	-	-	-	-	-	-	-
	Dominant	0.226	-0.20	0.16	-	-	-	-	-	-	-	-	-
	Recessive	0.195	-0.23	0.17	-	-	-	-	-	-	-	-	-
rs728488	Additive	0.191	-0.15	0.11	-	-	-	-	-	-	-	-	-
	Dominant	0.103	-0.38	0.22	-	-	-	-	-	-	-	-	-
	Recessive	0.471	-0.11	0.16	-	-	-	-	-	-	-	-	-
rs612399	Additive	0.063	-0.21	0.11	-	-	-	-	-	-	-	-	-
	Dominant	0.193	-0.20	0.15	-	-	-	-	-	-	-	-	-
	Recessive	0.098	-0.38	0.22	-	-	-	-	-	-	-	-	-
rs2234246	Additive	**0.003**	0.30	0.09	**0.0007**	0.13	0.03	0.874	0.02	0.17	**0.007**	0.49	0.16
	Dominant	**0.0003**	0.49	0.11	**0.001**	0.21	0.06	0.225	0.31	0.24	**0.002**	0.79	0.23
	Recessive	0.440	0.17	0.22	0.017	0.15	0.06	0.215	-0.40	0.31	0.250	0.42	0.35
rs2234237	Additive	0.337	-0.15	0.15	-	-	-	-	-	-	-	-	-
	Dominant	0.337	-0.15	0.15	-	-	-	-	-	-	-	-	-
	Recessive	-	-	-	-	-	-	-	-	-	-	-	-
rs13211886	Additive	0.953	0.01	0.19	-	-	-	-	-	-	-	-	-
	Dominant	0.953	0.01	0.19	-	-	-	-	-	-	-	-	-
	Recessive	-	-	-	-	-	-	-	-	-	-	-	-
rs6910730	Additive	0.337	-0.15	0.15	-	-	-	-	-	-	-	-	-
	Dominant	0.337	-0.15	0.15	-	-	-	-	-	-	-	-	-
	Recessive	-	-	-	-	-	-	-	-	-	-	-	-
rs9471554	Additive	0.054	0.28	0.13	-	-	-	-	-	-	-	-	-
	Dominant	0.054	0.28	0.13	-	-	-	-	-	-	-	-	-
	Recessive	-	-	-	-	-	-	-	-	-	-	-	-

**Fig 2:**
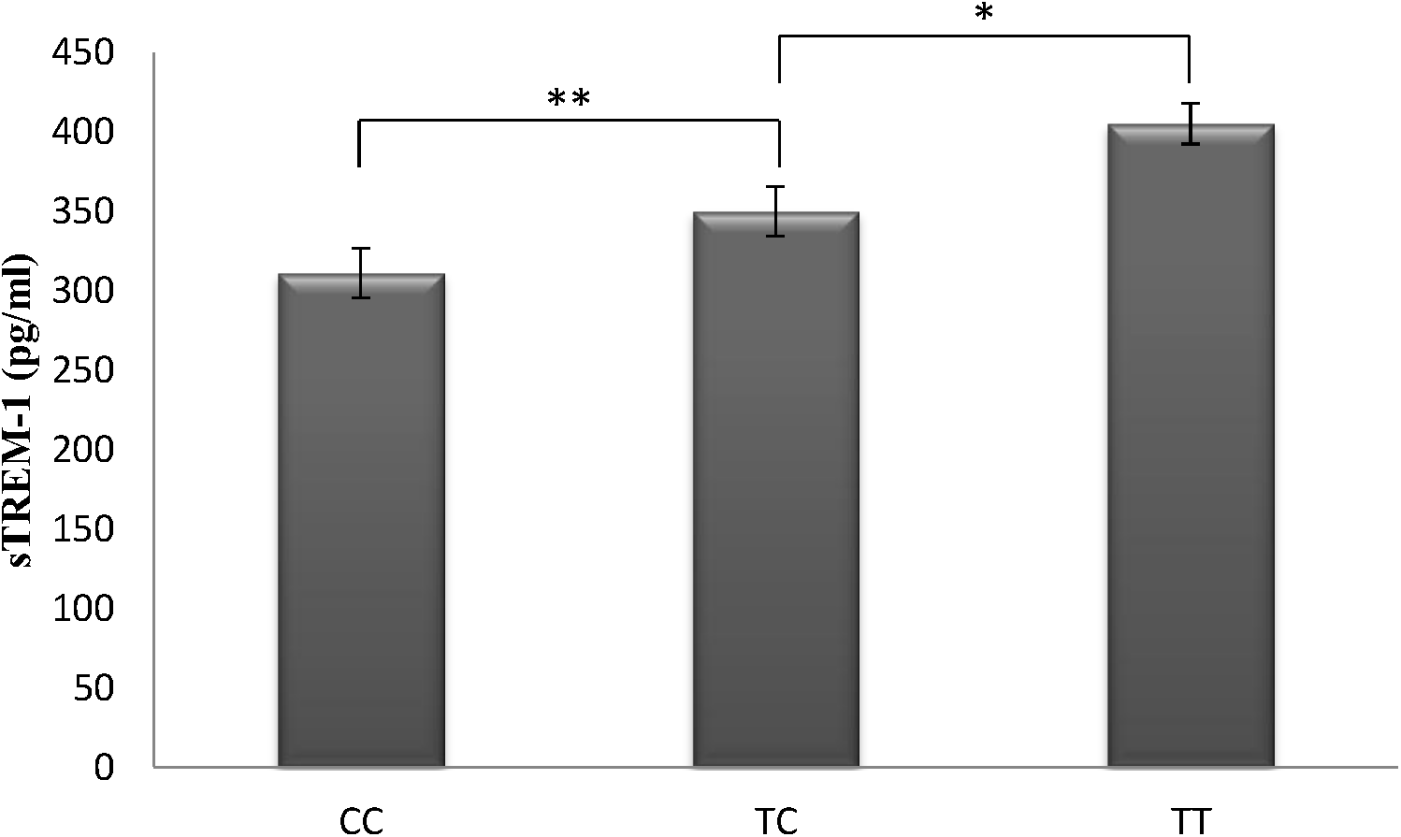
Mean values of sTREM-1 levels according to the different genotypes of rs2234246 (CC vs TC vs TT) in the replication population. Thin bars show standard errors. CC; Homozygous for the major allele of the rs2234246. TC; Heterozygous for the rs2234246. TT; Homozygous for the minor allele of the rs2234246. The significance between genotypes is showed as follows; N.S.; >0.05, * p<0.05, ** p<0.01, *** p<0.001.

The SNP rs2234246 was then genotyped in 80 additional and independent individuals (replication population). Additive and dominant models confirmed the association between the minor allele T and sTREM-1 levels (p-values = 0.0007 and 0.0017 respectively, β = 0.13 and 0.22 respectively, data available in Table 3 and Fig 2). Threshold of significance was 0.05 (as only 1 SNP was tested). The TREM-1 variance explained by the model is of 13% in the replication population.

The serum TREM-1sv levels, generated by the mRNA splicing variant TREM-1sv, were also measured in 78 individuals of the replication population. The TREM-1sv levels were detected only in four of the 78 samples studied, allowing no statistical analysis.

### Association studies between the rs2234246 in *TREM-1* and mRNA expression levels (mbTREM-1 and TREM-1sv)

Association analyses were then performed between the rs2234246 SNP and the expression levels of the two different alternative splicing isoforms of *TREM-1* (mbTREM-1 and TREM-1sv mRNAs) in PBMCs of the 30 individuals of the discovery population (threshold of significance was 0.05/1 SNP/2 phenotypes = 0.025). Interestingly, the SNP rs2234246 showed significant association with increased mRNA levels of the splicing form that codes for mbTREM-1 (additive model, p-value = 0.007, β = 0.49, data available on Table 3), whereas it was not associated with the levels of mRNA coding TREM-1sv. The TREM-1sv protein was only present in four samples out of the 78 samples studied, not showing increased expression in the presence of the SNP rs2234246.

### Association studies of the rs2234246 polymorphism in *TREM-1* with soluble selectins’ levels

The SNP rs2234246 was further genotyped in 351 individuals (discovery population, characteristics in Table 2). Association analyses were performed between the SNP rs2234246 and the sL-, sP- and sE-selectin levels (cut-off value for significance was set to 0.05/1 SNP/3 phenotypes = 0.016). Only the association with sL-selectin was significant (p-value = 0.011, β = 0.05) and explained a total of 2.1% of the variability of the sL-selectin. The minor allele T of the polymorphism was significantly associated with increased sL-selectin levels. No significant association was observed for sP- and sE-selectin levels.

The association between the minor allele T and sL-selectin levels was then confirmed in the 80 individuals of the replication population (p-value = 0.018, β = 0.03) (cut-off value for significance was set to 0.05, as only 1 SNP was tested) and association explained a total of 4.3% of the variability of the sL-selectin. All results are available in the Table 4.

**Table 4:** Effects of the polymorphism rs2234246 located within the *TREM-1* gene on the serum levels of the studied selectin molecules. MAF = Minor allele frequency of the rs2234246. Cutoff value of significance: 0.016 in discovery and 0.05 in replication population.

Population	Phenotypes	β	S.E	P-value
**Discovery population** **(MAF: 0.498)**	sL-selectin (mg/l) (n = 351)	0.05	0.02	**0.011**
	sP-selectin (mg/l) (n = 311)	0.02	0.02	0.435
	sE-selectin (mg/l) (n = 351)	0.01	0.04	0.685
**Replication population** **(MAF: 0.487)**	sL-selectin (mg/l) (n = 80)	0.03	0.01	**0.018**

### Regulatory environment of *TREM-1* rs2234246

Using bioinformatics analyses, we established the regulatory environment of SNP rs2234246 (S1 Fig). The rs2234246 polymorphism is located at 41276002 bp on the forward strand. It is positioned halfway between an open chromatin zone and a promoter flanking region. We can note that the open chromatin zone is active only in monocytes CD14+, which are present in PBMCs. Furthermore, the promoter flanking region is in an active state only in two cell types: the monocytes CD14+ and CD14+CD16-. It can be observed as well that the polymorphism is located in the 3’UTR region of the two mRNA splicing variants studied: mbTREM-1 (TREM1-001) and TREM-1sv (TREM1-002).

It is important to mention also that in the non-leukocyte cell types, the neighbourhood of *TREM-1* gene show only repressed or inactive areas, suggesting that these areas are not subject to regulations (results not shown).

### Epigenetic footprint of *TREM-1* rs2234246

Using bioinformatics tools, we established the epigenetic profiles (methylation / acetylation) of rs2234246 according to the cell type involved (S2 Fig). A specific epigenetic pattern emerged in monocytes (CD14+ and CD14+CD16-), vein blood neutrophils, eosinophils and macrophages in particular by the presence of H3K36me3 and H3K4me1. This specific methylation pattern is not present in the other 18 cell types investigated. Especially the presence of H3K36me3 is interesting, as is considered as a hallmark of actively transcribed regions. Thus, we speculate that the genetic region where the SNP rs2234246 is located is prone of higher expression levels of the *TREM-1* gene.

### Potential transcription factor binding sites in the rs2234246 locus

The bioinformatics results showed also differences in the potential transcriptional factors binding the locus according to the different alleles of the SNP rs2234246.

**Fig 3:**
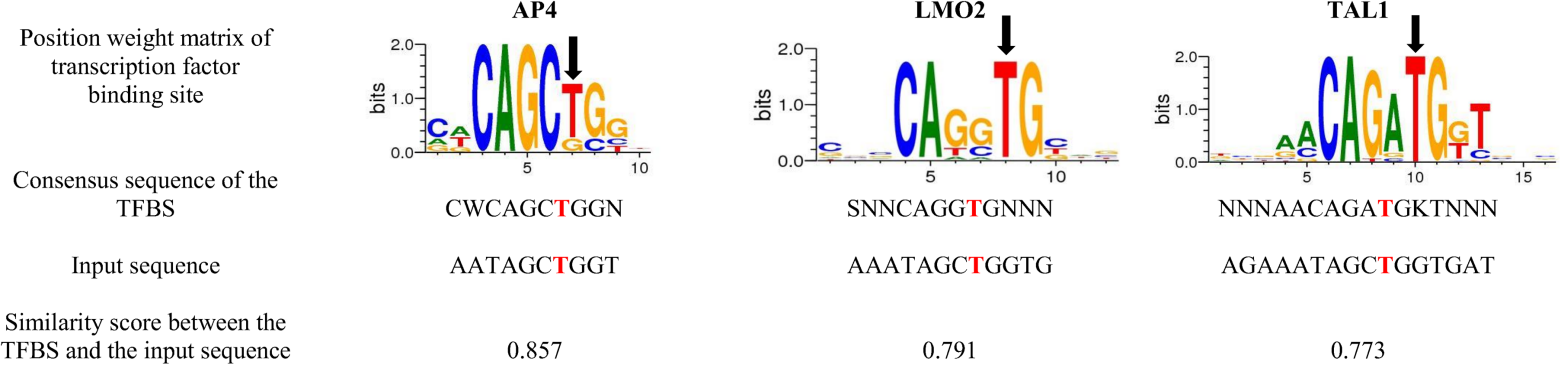
Specific transcription factor binding sites for the minor allele T of the SNP rs2234246.

When the minor allele is present (T), the specific potential transcription factors that are able to bind are AP4, LMO2 and TAL1, with a similarity score of 0.857, 0.791 and 0.773, respectively. Regarding the position weight matrix, we can see that the minor allele T is a highly conserved nucleotide in the TAL1 binding site, having a height of 2.0 bits. In the case of the transcription factor binding site LMO2 and AP4, the T allele is also important with a height >1.7 and >1.2 bits, respectively (Fig 3).

**Fig 4:**
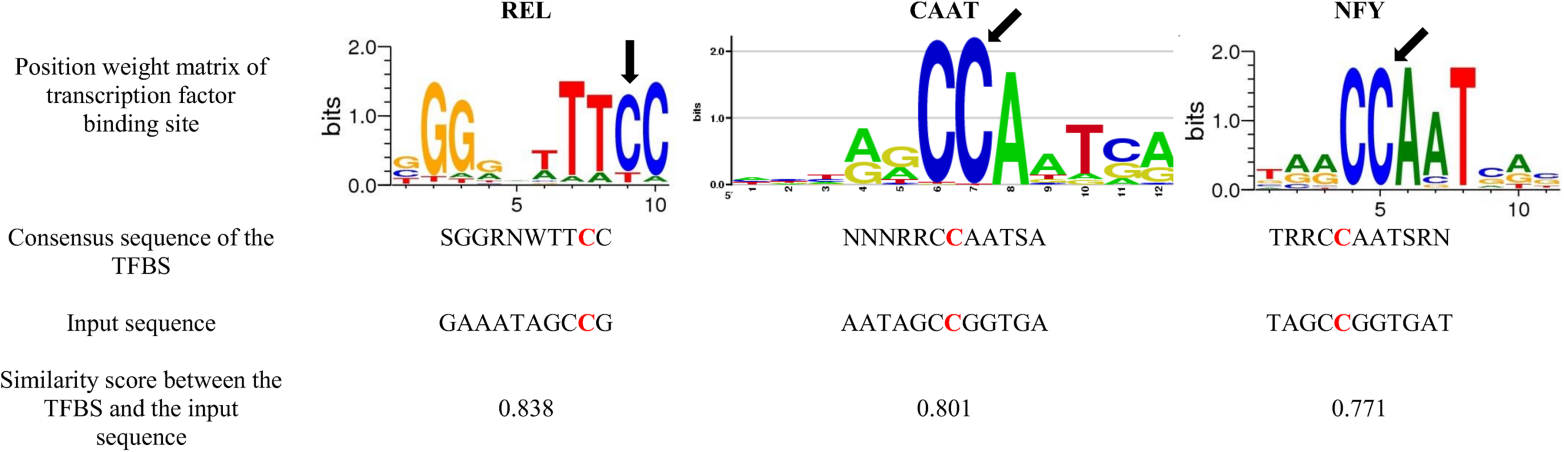
Specific transcription factor binding sites for the major allele C of the SNP rs2234246. The consensus sequence (fixed) of the transcription factor binding sites means: S = C or G, W = A or T, R = A or G, Y = C or T, K = G or T, M = A or C, N = any base pair. The input sequence was 38pb length, centred on the SNP of interest rs2234246. Only TFBS harbouring a nucleotide in its consensus sequence in coherence with the SNP of interest were selected.

When the major allele is present (C), the specific potential transcription factors that are able to bind are REL, CAAT and NFY, showing a similarity score of 0.838, 0.801 and 0.771 respectively. The position weight matrix shows that the minor allele C is highly conserved in the case of the CAAT and NFY binding sites (height >2 bits and >1.7 bits, respectively). In the binding site of REL, the C allele is also a conserved nucleotide with a height >1.2 bits (Fig 4).

## Discussion

TREM-1 protein levels have been related to numerous diseases where inflammation and inflammatory cell extravasation play central roles, such as atherosclerosis [10], acute myocardial infarction [11], and critical limb ischemia [12]. All these diseases have an important genetic component [34, 35], and despite all advances in treatment and prevention, they remain the leading cause of death worldwide [36]. This makes necessary the detection of genetic polymorphisms that could uncover novel metabolic pathways involved in the pathophysiology of these diseases, and therefore, improve their prevention and treatment [37, 38].

After a bibliography search of the SNPs within or near *TREM-1*, a total of 10 polymorphisms were selected and further analysed. Among them, we found that rs2234246, located in the mRNA 3’UTR region of *TREM-1*, was associated with sTREM-1 protein levels. So far, this is the first polymorphism that has been reported to affect the sTREM-1 levels. The minor allele T of this polymorphism was strongly associated with increased sTREM-1 levels, both in the discovery and the replication populations (p-values = 0.003 and 0.0007 respectively), explaining a large percentage of its phenotypic variation (33% and 13% in the discovery and replication population respectively).

It has been widely reported that increased levels of sTREM-1 were correlated to the severity of above-mentioned inflammatory diseases and poor prognosis [8–10, 12, 14–18], and the most recent studies have showed that *TREM-1* deletion or blockade is associated with up to 60% reduction of the development of atherosclerosis [10]. Thus, the T allele of rs2234246 may be considered as a risk factor, while the C allele may be a protective factor for these diseases. Given the high frequency of the polymorphism in the general population (about 50%), this result could be of high interest in further personalized medicine strategies for stratifying patients according to their risk to the above pathologies. In this context, it is important to note that a previous study has associated the minor allele of the SNP rs2234246 with an increased 3.1 odds ratio for septic shock [39]. This finding strengthens our hypothesis on the utility of the polymorphisms located in *TREM-1* gene in risk stratification. Another SNP located also within the TREM-1 gene (rs4711668) has been associated with severe coronary atherosclerosis in a Russian population [27]. However, it is not known whether this polymorphism is affecting the TREM-1 protein levels and although it is close to our SNP of interest, the 2 SNPs are not in strong linkage disequilibrium between them (r^2^ = 0.46).

We have also associated the T allele of the SNP rs2234246 with the expression levels of mbTREM-1 mRNA in PBMCs (p-value = 0.007 β = 0.49). This result is conferring functional properties to this polymorphism. However, we didn’t differentiate the monocyte subsets, and the expression of TREM-1 as well as the effect of the SNP rs2234246 could vary according the different monocyte subtypes. It is important to note that according to our bioinformatics analysis, the polymorphism is located in the 3’UTR region of the two mRNA splicing variants studied (S1 Fig). Interestingly, the polymorphism rs2234246 was related to an increase in the expression level of the mRNA coding mbTREM-1, but it was not related to the mRNA that codes TREM-1sv, nor to the TREM-1sv protein levels itself, which was not present in the serum. Two hypotheses have been proposed for the origin of soluble TREM-1: (1) splicing of different variants of alternative mRNA, which generates the TREM-1sv [40] and (2) shedding of mbTREM-1 by metalloproteases, which generates the sTREM-1. Our results, taking also into consideration that TREM-1sv protein levels were not present in the serum, support the hypothesis that the levels of sTREM-1 are controlled post transcriptionally by metalloproteases, rather than by alternatively spliced forms of RNA [7, 29].

According to our bioinformatics-epigenetics results, the rs2234246 has a specific H3K36me3 and H3K4me1 methylation epigenetic patterns in two groups of monocytes (CD14+ and CD14+CD16-), which are present in PBMCs. The presence of H3K36me3 is especially interesting as it is considered to be a hallmark of actively transcribed gene bodies. Thus, we speculate that the genetic region where the SNP rs2234246 is located, is prone of higher expression levels of the *TREM-1* gene [41]. However, this is only an assumption, as we were not able to perform experiments to confirm this effect. At the same time, the regulation profile of *TREM-1* shows an open chromatin zone and active promoter flanking regions only in CD14+ monocytes. The open chromatin zones are functionally related to transcriptional activity where DNA is accessible for the binding of proteins such as transcription factors [42]. Moreover, the SNP rs2234246, by the fact that it is located in the 3’-UTR of the mRNA, which is rich in regulatory regions that post-transcriptionally could influence gene expression [43, 44]. The effect of rs2234246 polymorphism on the sTREM-1 levels could be explained by several hypothetical mechanisms: (i) Post-transcriptional regulation of many pro-inflammatory mediators is controlled by adenosine and uridine-rich elements (AREs) [45]. AREs regions, located in the 3’-UTR of the mRNA can promote mRNA decay, affect mRNA stability, or activate translation. (ii) According to the TRANSFAC R.3.4. Database, the potential transcription factors binding the locus of the SNP rs2234246 could be different depending on the allele. The minor allele T is potentially associated with the matching of the transcription factors AP4, LMO2 and TAL1, while the major allele C is potentially associated with the matching of the transcription factors REL, CAAT and NFY. The type of transcription factors or even the affinity of those for the different polymorphisms of the rs2234246, could explain the changes in the expression levels of the *TREM-1* gene. However, we didn’t have experimental data that could confirm these possible direct affinity changes depending on the different alleles of the rs2234246, thus further experiments are needed to confirm this assumption.

It has been previously suggested that the receptors of the TREM family are regulating the cellular adhesion of macrophages and neutrophils *via* the phosphorylation of DAP12, which leads to activation of calcium sensitive kinases [6, 46]. At the same time, the number of studies supporting the importance of TREM-1 in the trans-epithelial migration of neutrophils and monocytes is increasing. Migrating neutrophils in septic patients have been found to bind to TREM-1 [7]. It has been previously demonstrated that TREM-1 is crucially involved in leukocyte recruitment after myocardial infarction and atherosclerosis [10, 11]. We demonstrated a five-fold decrease in the number of recruited neutrophils when TREM-1 was inhibited pharmacologically [11]. Also, in acute respiratory infections, TREM-1 is required for the trans-epithelial migration of neutrophils into the lung [13]. Despite these arguments, so far, the process by which TREM-1 contributes to this trans-epithelial migration is unknown. In our study, we demonstrated for the first time that TREM-1 is regulating one adhesion molecule.

We found that the rs2234246 polymorphism is specifically correlated with increased plasma levels of L -selectin. The soluble L-selectin levels are thought to represent a homeostatic effort to limit excessive inflammation. Indeed, they are correlated with the severity of inflammatory diseases, including cardio-vascular diseases [47, 48]. Although, once in a soluble form, they may have reduced adhesion, migration and trans-epithelial migration capacity, it can be postulated that TREM-1 increases inflammation in general, leading to L-selectin shedding as a downstream effect. Especially, L-selectin, has been showed to be important in the recruitment of monocytes and neutrophils to sites of acute and chronic inflammation [49]. The role of the selectins in inflammatory processes is well established, and the selectin-mediated adhesion and signalling contribute to different cardio-vascular diseases [21]. The selectins, have been shown to contribute to atherosclerosis [24, 25] and arterial thrombosis [50]. According to our results, the minor allele T of the rs2234246 could act as a risk factor as it is correlated with an increase of 2.1–4.3% of sL-selectin [47, 48, 51]. It would be also interesting to further investigate the possible associations between the membrane bound L-selectin levels and rs2234246. Unfortunately, in this work we were not able to address this issue.

The conclusion from our bioinformatics and transcriptomic analyses supports the hypothesis that TREM-1 is involved in the trans-epithelial migration process of leukocytes, more specifically of monocytes, that could be effective through a higher level of inflammation, which could be observed with an overexpression of sL-selectin.

One of the advantages of our research is that we limited the potential confounders by using a homogeneous population coming from the region of Lorraine in France as a discovery population. Because of this, we have been able to reduce the enormous cardio-vascular-related heterogeneity of the population, by linking genes of interest to intermediate phenotypes and not to the disease. This approach can represent better the real biological pathways where the gene of interest is involved. Moreover, we replicated the results in an independent population, while simultaneously bioinformatics and bibliographical data also strengthen our results.

One limitation of our study is that we were not able to realize gene expression analysis in the PMNs and more specifically in neutrophils. As well as the monocytes, the neutrophils are also expressing TREM-1 protein. However, we were not able to have direct experimental data about the effect of the SNP rs2234246 in neutrophils due to lack of biological material. Future studies are needed to confirm these possible effects. Also, we couldn’t make the distinction among the different subgroup of monocytes and TREM-1 expression, and the effect of the SNP rs2234246 may be different in those subgroups. A more specific study including the monocyte subsets would significantly improve the biological relevance of the SNP rs2234246. In conclusion, our study led to the discovery of one polymorphism (rs2234246) strongly affecting sTREM-1 protein levels and associated to an increase in the levels of the mRNA coding mbTREM-1 in PBMCs. Since no association was established with splicing mRNA and TREM-1sv was not detected in the serum of the individuals, it seems that, the levels of sTREM-1 are controlled post transcriptionally by metalloproteases. Interestingly, we demonstrated for the first time that *TREM-1* rs2234246 polymorphism can also modulate the sL-selectin levels via a higher inflammatory state, suggesting that TREM-1 acts in the trans-epithelial migration process of leukocytes and more specifically of monocytes through expression of sL-selectin.

## Supporting information

S1 Fig Regulation profile of the *TREM-1* gene in different cell types expressing the protein TREM-1. The polymorphism rs2234246 is located at 41276002 bp on the forward strand (vertical red line). It is positioned halfway between an open chromatin zone and a promoter flanking region. It can also be observed that the polymorphism is located in the 3’UTR region of the two mRNA splicing variants studied: mbTREM-1 (TREM1-001; ENST00000244709.8) and TREM-1sv (TREM1-002; ENST00000334475.10) and in an intron zone of another *TREM-1* transcript: TREM1-006 (ENST00000589695.1) (VB: Vein blood).
(DOCX)

S2 Fig Epigenetic profile of rs2234246 in leukocytes cell types: Specific histone methylation patterns (VB: Vein blood). (DOCX)

## References

[1] Drechsler M, Megens RT, van Zandvoort M, Weber C, Soehnlein O. Hyperlipidemia-triggered neutrophilia promotes early atherosclerosis. Circulation. 2010;122(18):1837–45. doi: 10.1161/CIRCULATIONAHA.110.961714 20956207 10.1161/CIRCULATIONAHA.110.961714

[2] Weber C, Noels H. Atherosclerosis: current pathogenesis and therapeutic options. Nat Med. 2011;17(11):1410–22. doi: 10.1038/nm.2538 22064431 10.1038/nm.2538

[3] Bouchon A, Dietrich J, Colonna M. Cutting edge: inflammatory responses can be triggered by TREM-1, a novel receptor expressed on neutrophils and monocytes. J Immunol. 2000;164(10):4991–5. 10799849 10.4049/jimmunol.164.10.4991

[4] Bouchon A, Facchetti F, Weigand MA, Colonna M. TREM-1 amplifies inflammation and is a crucial mediator of septic shock. Nature. 2001;410(6832):1103–7. doi: 10.1038/35074114 11323674 10.1038/35074114

[5] Colonna M, Facchetti F. TREM-1 (triggering receptor expressed on myeloid cells): a new player in acute inflammatory responses. J Infect Dis. 2003;187 Suppl 2:S397–401. 12792857 10.1086/374754

[6] Ford JW, McVicar DW. TREM and TREM-like receptors in inflammation and disease. Curr Opin Immunol. 2009;21(1):38–46. doi: 10.1016/j.coi.2009.01.009 19230638 10.1016/j.coi.2009.01.009 PMC2723941

[7] Wong-Baeza I, Gonzalez-Roldan N, Ferat-Osorio E, Esquivel-Callejas N, Aduna-Vicente R, Arriaga-Pizano L, et al Triggering receptor expressed on myeloid cells (TREM-1) is regulated post-transcriptionally and its ligand is present in the sera of some septic patients. Clin Exp Immunol. 2006;145(3):448–55. doi: 10.1111/j.1365-2249.2006.03158.x 16907912 10.1111/j.1365-2249.2006.03158.x PMC1809719

[8] Boufenzer A, Lemarie J, Simon T, Derive M, Bouazza Y, Tran N, et al TREM-1 Mediates Inflammatory Injury and Cardiac Remodeling Following Myocardial Infarction. Circ Res. 2015;116(11):1772–82. doi: 10.1161/CIRCRESAHA.116.305628 25840803 10.1161/CIRCRESAHA.116.305628

[9] Kuai J, Gregory B, Hill A, Pittman DD, Feldman JL, Brown T, et al TREM-1 expression is increased in the synovium of rheumatoid arthritis patients and induces the expression of pro-inflammatory cytokines. Rheumatology (Oxford). 2009;48(11):1352–8. 19713442 10.1093/rheumatology/kep235

[10] Joffre J, Potteaux S, Zeboudj L, Loyer X, Boufenzer A, Laurans L, et al Genetic and Pharmacological Inhibition of TREM-1 Limits the Development of Experimental Atherosclerosis. J Am Coll Cardiol. 2016;68(25):2776–93. doi: 10.1016/j.jacc.2016.10.015 28007141 10.1016/j.jacc.2016.10.015

[11] Jeremie L, Amir B, Marc D, Sebastien G. The Triggering Receptor Expressed on Myeloid cells-1: A new player during acute myocardial infarction. Pharmacol Res. 2015;100:261–5. doi: 10.1016/j.phrs.2015.07.027 26318764 10.1016/j.phrs.2015.07.027

[12] Dopheide JF, Doppler C, Scheer M, Obst V, Radmacher MC, Radsak MP, et al Critical limb ischaemia is characterised by an increased production of whole blood reactive oxygen species and expression of TREM-1 on neutrophils. Atherosclerosis. 2013;229(2):396–403. doi: 10.1016/j.atherosclerosis.2013.05.029 23880194 10.1016/j.atherosclerosis.2013.05.029

[13] Klesney-Tait J, Keck K, Li X, Gilfillan S, Otero K, Baruah S, et al Transepithelial migration of neutrophils into the lung requires TREM-1. J Clin Invest. 2013;123(1):138–49. doi: 10.1172/JCI64181 23241959 10.1172/JCI64181 PMC3533287

[14] Altay FA, Elaldi N, Cicek Senturk G, Altin N, Gozel MG, Albayrak Y, et al Serum sTREM-1 level is quite higher in Crimean Congo Hemorrhagic Fever, a viral infection. J Med Virol. 2016. 10.1002/jmv.24496 26877157

[15] Radsak MP, Taube C, Haselmayer P, Tenzer S, Salih HR, Wiewrodt R, et al Soluble triggering receptor expressed on myeloid cells 1 is released in patients with stable chronic obstructive pulmonary disease. Clin Dev Immunol. 2007;2007:52040 doi: 10.1155/2007/52040 18317529 10.1155/2007/52040 PMC2246041

[16] Yasuda T, Takeyama Y, Ueda T, Shinzeki M, Sawa H, Takahiro N, et al Increased levels of soluble triggering receptor expressed on myeloid cells-1 in patients with acute pancreatitis. Crit Care Med. 2008;36(7):2048–53. doi: 10.1097/CCM.0b013e31817b8824 18552693 10.1097/CCM.0b013e31817b8824

[17] Tejera A, Santolaria F, Diez ML, Aleman-Valls MR, Gonzalez-Reimers E, Martinez-Riera A, et al Prognosis of community acquired pneumonia (CAP): value of triggering receptor expressed on myeloid cells-1 (TREM-1) and other mediators of the inflammatory response. Cytokine. 2007;38(3):117–23. doi: 10.1016/j.cyto.2007.05.002 17659879 10.1016/j.cyto.2007.05.002

[18] Park JJ, Cheon JH, Kim BY, Kim DH, Kim ES, Kim TI, et al Correlation of serum-soluble triggering receptor expressed on myeloid cells-1 with clinical disease activity in inflammatory bowel disease. Dig Dis Sci. 2009;54(7):1525–31. doi: 10.1007/s10620-008-0514-5 18975078 10.1007/s10620-008-0514-5

[19] Derive M, Bouazza Y, Sennoun N, Marchionni S, Quigley L, Washington V, et al Soluble TREM-like transcript-1 regulates leukocyte activation and controls microbial sepsis. J Immunol. 2012;188(11):5585–92. doi: 10.4049/jimmunol.1102674 22551551 10.4049/jimmunol.1102674 PMC6382278

[20] Derive M, Boufenzer A, Bouazza Y, Groubatch F, Alauzet C, Barraud D, et al Effects of a TREM-like transcript 1-derived peptide during hypodynamic septic shock in pigs. Shock. 2013;39(2):176–82. doi: 10.1097/SHK.0b013e31827bcdfb 23324887 10.1097/SHK.0b013e31827bcdfb

[21] McEver RP. Selectins: initiators of leucocyte adhesion and signalling at the vascular wall. Cardiovasc Res. 2015;107(3):331–9. doi: 10.1093/cvr/cvv154 25994174 10.1093/cvr/cvv154 PMC4592324

[22] Zemans RL, Colgan SP, Downey GP. Transepithelial migration of neutrophils: mechanisms and implications for acute lung injury. Am J Respir Cell Mol Biol. 2009;40(5):519–35. doi: 10.1165/rcmb.2008-0348TR 18978300 10.1165/rcmb.2008-0348TR PMC2677434

[23] Muller WA. The regulation of transendothelial migration: new knowledge and new questions. Cardiovasc Res. 2015;107(3):310–20. doi: 10.1093/cvr/cvv145 25987544 10.1093/cvr/cvv145 PMC4592322

[24] An G, Wang H, Tang R, Yago T, McDaniel JM, McGee S, et al P-selectin glycoprotein ligand-1 is highly expressed on Ly-6Chi monocytes and a major determinant for Ly-6Chi monocyte recruitment to sites of atherosclerosis in mice. Circulation. 2008;117(25):3227–37. doi: 10.1161/CIRCULATIONAHA.108.771048 18519846 10.1161/CIRCULATIONAHA.108.771048 PMC2596619

[25] Dong ZM, Chapman SM, Brown AA, Frenette PS, Hynes RO, Wagner DD. The combined role of P- and E-selectins in atherosclerosis. J Clin Invest. 1998;102(1):145–52. doi: 10.1172/JCI3001 9649568 10.1172/JCI3001 PMC509076

[26] Blankenberg S, Barbaux S, Tiret L. Adhesion molecules and atherosclerosis. Atherosclerosis. 2003;170(2):191–203. 14612198 10.1016/s0021-9150(03)00097-2

[27] Kutikhin AG, Ponasenko AV, Khutornaya MV, Yuzhalin AE, Zhidkova II, Salakhov RR, et al Association of TLR and TREM-1 gene polymorphisms with atherosclerosis severity in a Russian population. Meta Gene. 2016;9:76–89. doi: 10.1016/j.mgene.2016.04.001 27200266 10.1016/j.mgene.2016.04.001 PMC4864274

[28] Baruah S, Keck K, Vrenios M, Pope MR, Pearl M, Doerschug K, et al Identification of a Novel Splice Variant Isoform of TREM-1 in Human Neutrophil Granules. J Immunol. 2015;195(12):5725–31. doi: 10.4049/jimmunol.1402713 26561551 10.4049/jimmunol.1402713 PMC4670805

[29] Gomez-Pina V, Soares-Schanoski A, Rodriguez-Rojas A, Del Fresno C, Garcia F, Vallejo-Cremades MT, et al Metalloproteinases shed TREM-1 ectodomain from lipopolysaccharide-stimulated human monocytes. J Immunol. 2007;179(6):4065–73. 17785845 10.4049/jimmunol.179.6.4065

[30] Visvikis-Siest S, Siest G. The STANISLAS Cohort: a 10-year follow-up of supposed healthy families. Gene-environment interactions, reference values and evaluation of biomarkers in prevention of cardiovascular diseases. Clin Chem Lab Med. 2008;46(6):733–47. doi: 10.1515/CCLM.2008.178 18601594 10.1515/CCLM.2008.178

[31] Aken BL, Achuthan P, Akanni W, Amode MR, Bernsdorff F, Bhai J, et al Ensembl 2017. Nucleic Acids Res. 2017;45(D1):D635–D42. doi: 10.1093/nar/gkw1104 27899575 10.1093/nar/gkw1104 PMC5210575

[32] Matys V, Fricke E, Geffers R, Gossling E, Haubrock M, Hehl R, et al TRANSFAC: transcriptional regulation, from patterns to profiles. Nucleic acids research. 2003;31(1):374–8. 12520026 10.1093/nar/gkg108 PMC165555

[33] Purcell S, Neale B, Todd-Brown K, Thomas L, Ferreira MA, Bender D, et al PLINK: a tool set for whole-genome association and population-based linkage analyses. Am J Hum Genet. 2007;81(3):559–75. doi: 10.1086/519795 17701901 10.1086/519795 PMC1950838

[34] Arnett DK, Baird AE, Barkley RA, Basson CT, Boerwinkle E, Ganesh SK, et al Relevance of genetics and genomics for prevention and treatment of cardiovascular disease: a scientific statement from the American Heart Association Council on Epidemiology and Prevention, the Stroke Council, and the Functional Genomics and Translational Biology Interdisciplinary Working Group. Circulation. 2007;115(22):2878–901. doi: 10.1161/CIRCULATIONAHA.107.183679 17515457 10.1161/CIRCULATIONAHA.107.183679

[35] Cambien F, Tiret L. Genetics of cardiovascular diseases: from single mutations to the whole genome. Circulation. 2007;116(15):1714–24. doi: 10.1161/CIRCULATIONAHA.106.661751 17923582 10.1161/CIRCULATIONAHA.106.661751

[36] Mozaffarian D, Benjamin EJ, Go AS, Arnett DK, Blaha MJ, Cushman M, et al Heart disease and stroke statistics—2015 update: a report from the American Heart Association. Circulation. 2015;131(4):e29–322. doi: 10.1161/CIR.0000000000000152 25520374 10.1161/CIR.0000000000000152

[37] Swerdlow DI, Hingorani AD, Humphries SE. Genetic risk factors and Mendelian randomization in cardiovascular disease. Curr Cardiol Rep. 2015;17(5):33 doi: 10.1007/s11886-015-0584-x 25894797 10.1007/s11886-015-0584-x

[38] Gerszten RE, Wang TJ. The search for new cardiovascular biomarkers. Nature. 2008;451(7181):949–52. doi: 10.1038/nature06802 18288185 10.1038/nature06802

[39] Peng LS, Li J, Zhou GS, Deng LH, Yao HG. Relationships between genetic polymorphisms of triggering receptor expressed on myeloid cells-1 and septic shock in a Chinese Han population. World J Emerg Med. 2015;6(2):123–30. doi: 10.5847/wjem.j.1920-8642.2015.02.007 26056543 10.5847/wjem.j.1920-8642.2015.02.007 PMC4458472

[40] Klesney-Tait J, Turnbull IR, Colonna M. The TREM receptor family and signal integration. Nat Immunol. 2006;7(12):1266–73. doi: 10.1038/ni1411 17110943 10.1038/ni1411

[41] Kolasinska-Zwierz P, Down T, Latorre I, Liu T, Liu XS, Ahringer J. Differential chromatin marking of introns and expressed exons by H3K36me3. Nat Genet. 2009;41(3):376–81. doi: 10.1038/ng.322 19182803 10.1038/ng.322 PMC2648722

[42] Heintzman ND, Stuart RK, Hon G, Fu Y, Ching CW, Hawkins RD, et al Distinct and predictive chromatin signatures of transcriptional promoters and enhancers in the human genome. Nat Genet. 2007;39(3):311–8. doi: 10.1038/ng1966 17277777 10.1038/ng1966

[43] Pichon X, Wilson LA, Stoneley M, Bastide A, King HA, Somers J, et al RNA binding protein/RNA element interactions and the control of translation. Curr Protein Pept Sci. 2012;13(4):294–304. doi: 10.2174/138920312801619475 22708490 10.2174/138920312801619475 PMC3431537

[44] Barrett LW, Fletcher S, Wilton SD. Regulation of eukaryotic gene expression by the untranslated gene regions and other non-coding elements. Cell Mol Life Sci. 2012;69(21):3613–34. doi: 10.1007/s00018-012-0990-9 22538991 10.1007/s00018-012-0990-9 PMC3474909

[45] Chen CY, Shyu AB. AU-rich elements: characterization and importance in mRNA degradation. Trends Biochem Sci. 1995;20(11):465–70. 8578590 10.1016/s0968-0004(00)89102-1

[46] Mocsai A, Abram CL, Jakus Z, Hu Y, Lanier LL, Lowell CA. Integrin signaling in neutrophils and macrophages uses adaptors containing immunoreceptor tyrosine-based activation motifs. Nat Immunol. 2006;7(12):1326–33. doi: 10.1038/ni1407 17086186 10.1038/ni1407 PMC4698344

[47] Siminiak T, Smielecki J, Dye JF, Balinski M, El-Gendi H, Wysocki H, et al Increased release of the soluble form of the adhesion molecules L-selectin and ICAM-1 but not E-selectin during attacks of angina pectoris. Heart Vessels. 1998;13(4):189–94. 10442400 10.1007/BF01745043

[48] Albertini JP, Valensi P, Lormeau B, Vaysse J, Attali JR, Gattegno L. Soluble L-selectin level is a marker for coronary artery disease in type 2 diabetic patients. Diabetes Care. 1999;22(12):2044–8. 10587840 10.2337/diacare.22.12.2044

[49] Ivetic A. Signals regulating L-selectin-dependent leucocyte adhesion and transmigration. Int J Biochem Cell Biol. 2013;45(3):550–5. doi: 10.1016/j.biocel.2012.12.023 23299028 10.1016/j.biocel.2012.12.023

[50] Falati S, Liu Q, Gross P, Merrill-Skoloff G, Chou J, Vandendries E, et al Accumulation of tissue factor into developing thrombi in vivo is dependent upon microparticle P-selectin glycoprotein ligand 1 and platelet P-selectin. J Exp Med. 2003;197(11):1585–98. doi: 10.1084/jem.20021868 12782720 10.1084/jem.20021868 PMC2193915

[51] Wallberg-Jonsson S, Caidahl K, Klintland N, Nyberg G, Rantapaa-Dahlqvist S. Increased arterial stiffness and indication of endothelial dysfunction in long-standing rheumatoid arthritis. Scand J Rheumatol. 2008;37(1):1–5. doi: 10.1080/03009740701633238 18189187 10.1080/03009740701633238

