# Supplementary material for: TREM-1 SNP rs2234246 regulates TREM-1 protein and mRNA levels and is associated with plasma levels of L-selectin
Source: PLoS One. 2017 Aug 3;12(8):e0182226. doi: 10.1371/journal.pone.0182226 (PMC5542552; doi:10.1371/journal.pone.0182226)
Supplement: S1 Fig — The polymorphism rs2234246 is located at 41276002 bp on the forward strand (vertical red line). It is positioned halfway between an open chromatin zone and a promoter flanking region. It can also be observed that the polymorphism is located in the 3’UTR region of the two mRNA splicing variants studied: mbTREM-1 (TREM1-001; ENST00000244709.8) and TREM-1sv (TREM1-002; ENST00000334475.10) and in an intron zone of another TREM-1 transcript: TREM1-006 (ENST00000589695.1) (VB: Vein blood). (DOCX) [file pone.0182226.s001.docx]

**Supplementary Figure 1.** Regulation profile of the *TREM-1* gene in different cell types expressing the protein TREM-1. The polymorphism rs2234246 is located at 41276002 bp on the forward strand (vertical red line). It is positioned halfway between an open chromatin zone and a promoter flanking region. It can also be observed that the polymorphism is located in the 3’UTR region of the two mRNA splicing variants studied: mbTREM-1 (TREM1-001; ENST00000244709.8) and TREM-1sv (TREM1-002; ENST00000334475.10) and in an intron zone of another *TREM-1* transcript: TREM1-006 (ENST00000589695.1) (VB: Vein blood).

**
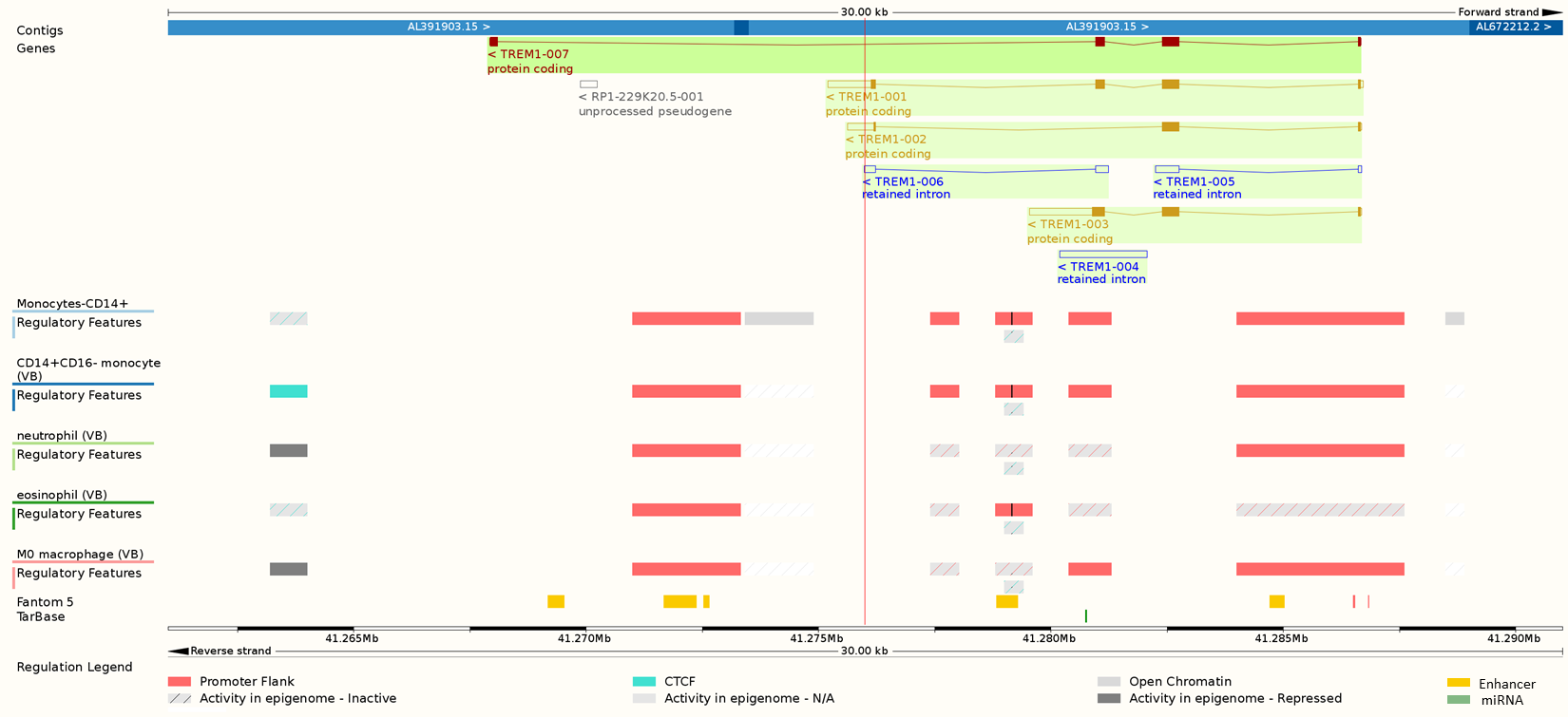
**
