# Supplementary material for: TREM-1 SNP rs2234246 regulates TREM-1 protein and mRNA levels and is associated with plasma levels of L-selectin
Source: PLoS One. 2017 Aug 3;12(8):e0182226. doi: 10.1371/journal.pone.0182226 (PMC5542552; doi:10.1371/journal.pone.0182226)
Supplement: S2 Fig — (DOCX) [file pone.0182226.s002.docx]

**Supplementary Figure 2. Epigenetic profile of rs2234246 in leukocytes cell types: specific histone methylation patterns (VB: Vein blood)**


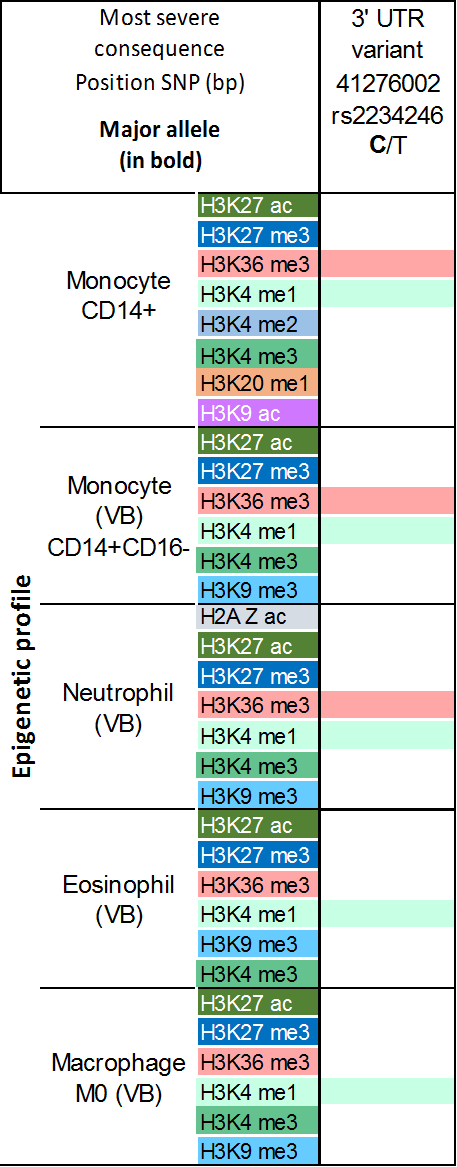


**List of cell types investigated:**

1. Monocytes CD14+
   1. CD14+ CD16- monocyte from venous blood
   2. CD14+ CD16- monocyte from cord blood
2. T cell

2.1 CD4+ ab T cell from venous blood

2.2 CD8+ ab T cell from cord blood

2.3 CM CD4+ ab T cell from venous blood

1. Human leukemic T-cell line with p53 (Notch) mutation
2. Endothelial progenitol cell (EPC) from venous blood
3. 5.1 GM12878 (Human B-lymphocyte cell line

5.2 Naïve B cell from venous blood

1. Human embryonic stem cells (H1ESC)
2. Human mammary epithelial cells (HMEC)
3. Skeletal muscle myotubes differenciated from the HSMM cell line
4. Human umbilical vein endothelial cell line (HUVEC)
5. Human epithelial carcinoma cells (HeLa)
6. Human hepatocellular liver carcinoma cell line (HepG2)
7. Normal human epidermal keratinocyte cell line (NHEK)
8. Human myelogenous leukaemia cell line (K562)
9. 14.1 M0 Macrophage from cord blood

14.2 M0 Macrophage from venous blood

14.3 M1 Macrophage from cord blood

14.4 M1 Macrophage from venous blood

14.5 M2 Macrophage from cord blood

14.6 M2 Macrophage from venous blood

1. Mesenchymal stem cells from venous blood (MSC)
2. Normal human astrocytes
3. 17.1 Human fetal lung fibroblast

17.2 Human adult dermal fibroblast

17.3 Human adult lung fibroblast

1. Osteoblasts (NHOst)
2. Eosinophil from venous blood
3. Neutro myelocyte from bone marrow
4. 21.1 Neutrophil from venous blood

21.2 Neutrophil from cord blood

1. Erythroblast from cord blood
